# Endothelial Progenitor Cells in Tumor Angiogenesis: Another Brick in the Wall

**DOI:** 10.1155/2015/832649

**Published:** 2015-04-27

**Authors:** Marina Marçola, Camila Eleuterio Rodrigues

**Affiliations:** ^1^Department of Physiology, Institute of Bioscience, University of São Paulo, Rua do Matão, Travessa 14, No. 101, Room 323, 05508-900 São Paulo, SP, Brazil; ^2^Medical Investigation Laboratory 12, School of Medicine, University of São Paulo, Avenida Dr. Arnaldo, No. 455, Room 3310, 01246-903 São Paulo, SP, Brazil

## Abstract

Until 15 years ago, vasculogenesis, the formation of new blood vessels from undifferentiated cells, was thought to occur only during embryonic development. The discovery of circulating cells that are able to promote vascular regeneration and repair—the so-called endothelial progenitor cells (EPCs)—changed that, and EPCs have since been studied extensively. It is already known that EPCs include many subtypes of cells that play a variety of roles in promoting vascular growth. Some EPCs are destined to differentiate into endothelial cells, whereas others are capable of promoting and sustaining angiogenesis through paracrine mechanisms. Vasculogenesis and angiogenesis might constitute complementary mechanisms for postnatal neovascularization, and EPCs could be at the core of this process. Although the formation of new blood vessels from preexisting vasculature plays a beneficial role in many physiological processes, such as wound healing, it also contributes to tumor growth and metastasis. However, many aspects of the role played by EPCs in tumor angiogenesis remain unclear. This review aims to address the main aspects of EPCs differentiation and certain characteristics of their main function, especially in tumor angiogenesis, as well as the potential clinical applications.

## 1. Introduction

In the past few years, a number of studies have shown that adult stem and progenitor cells play a role in tumor progression. Deregulation in the self-renewal programs of adult stem cells leads to cell transformation, contributing to the formation and development of new tumors [1]. Although angiogenesis (the formation of new blood vessels from preexisting vasculature) plays a beneficial role in many physiological processes, such as wound healing, it also contributes to the growth and metastasis of tumors.

Until the 1990s, postnatal neovascularization was thought to result from the detachment and proliferation of mature endothelial cells, supporting the idea that vasculogenesis (the formation of new blood vessels from progenitor cells or angioblasts) occurs only during embryogenesis. In 1997, Asahara et al. [2] isolated mononuclear cells from adult peripheral blood and found that those cells had the same characteristics as the embryonic angioblasts that contribute to the revascularization of ischemic tissue. In a subsequent study, Asahara et al. [3] coined the term “endothelial progenitor cells” (EPCs) to describe these cells. In that study, the authors showed that bone marrow-derived EPCs not only have therapeutic applications but also are involved in the pathological neovascularization of tumors and consequently in their growth. In 2004, Asahara and Kawamoto [4] proposed that vasculogenesis and angiogenesis constitute complementary mechanisms of postnatal neovascularization in which EPCs can play a role. More recently, studies have indicated that adult progenitor cells have the ability to migrate and proliferate, contributing to the* de novo* formation of capillary structures [5]. Therefore, EPCs have been defined as circulating progenitor cells that have the ability to differentiate and form functional blood vessels. However, the exact origin, character, and function of EPCs are still controversial in the literature, and their role in tumorigenesis is therefore also still under discussion. Here, we present the main issues involved in the characterization of EPCs and their role in angiogenesis, mainly in the promotion of tumor progression.

## 2. Characterization of EPCs

Human CD34^+^ cells isolated from circulating peripheral blood, umbilical cord blood, or bone marrow can differentiate into endothelial cells [2, 6], as well as being capable of contributing to neoendothelialization and neovascularization in the adult organism. These cells can promote angiogenesis by two different mechanisms [7–10]: serving as the substrate for new vessel formation and exerting a paracrine effect. In fact, there are two main cell types within the EPC designation [11–16]: early EPCs (angiogenic cells), which have features of hematopoietic cells, can generate monocytic cells, and play a role in vasculogenesis by secreting large quantities of angiogenic factors that act via paracrine mechanisms, and late EPCs (endothelial outgrowth cells), which are able to differentiate into endothelial cells and promote vascular tube formation.

**Figure 1:**
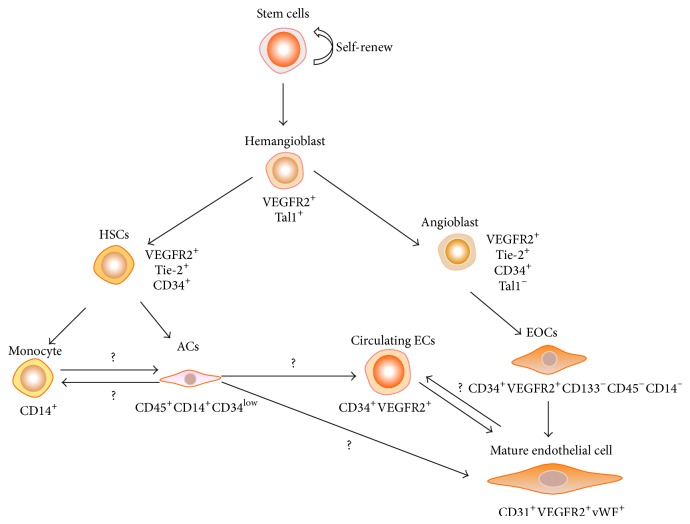
Adult endothelial progenitor cell phenotype (ACs: angiogenic cells; ECs: endothelial cells; EOCs: endothelial outgrowth cells; HSCs: hematopoietic stem cells; VEGFR: vascular endothelial growth factor receptor). Hemangioblasts derived from pluripotent stem cell can differentiate into HSCs and angioblasts. The HSCs give rise to blood cells, such as monocytes and ACs. Whether monocytes can act as ACs and vice versa is still controversial. Angioblasts give rise to endothelial cell lineage, including EOCs. Circulating endothelial cells can arise from the detachment of mature ECs and repair other areas of endothelium damage or can arise from the differentiation of ACs.

Although the functions of EPCs have been well described, their defining characteristics remain controversial in the literature. In general, EPCs have the ability to absorb acetylated low-density lipoprotein and to bind the lectin* Ulex europaeus* agglutinin I. Endothelial outgrowth cells differ from angiogenic cells due to their higher proliferative potential and their ability to promote the formation of vascular structures [12–16]. It is well known that, during hematoendothelial development, CD34^+^ cells do not express CD45, rather acquiring it during differentiation into hematopoietic progenitor cells, except if they are destined to differentiate into endothelial cells [7, 17–19]. Moreover, CD34 antigen has its expression gradually reduced as the level of maturation of hematopoietic cell lineages increases [20]. Therefore, CD45 and CD14 are mainstream antigens able to differentiate these cell types (Table 1), because endothelial outgrowth cells originate from CD34^+^ cells that are negative for CD45 and CD14, whereas angiogenic cells are CD45^+^/CD14^+^/CD34^low^ cells (Figure 1).

Classically, surface immunophenotyping of EPCs was expected to express CD34, vascular endothelial growth factor receptor-2 (VEGFR-2), and prominin 1 (CD133) [4]. However, some studies have suggested that CD34^+^/VEGFR-2^+^/CD133^+^ cells constitute an enriched population of CD45^+^ hematopoietic precursors, or even mature circulating endothelial cells, and therefore do not contribute to the formation of endothelial cells* in vitro* [7, 21, 22]. In addition, stem/progenitor cells of other origins are also capable of differentiating into endothelial cells and exist either in the bone marrow—including CD34^−^/CD133^+^ multipotent adult progenitor cells [23, 24], mesodermal progenitor cells [25], and side population cells [26]—or in the peripheral blood—including circulating endothelial precursors that can be derived from stem/progenitor cells in bone marrow or can arise by detachment of mature endothelial cells or perivascular cells, such as pericytes [27]. Furthermore, EPCs can express certain endothelial markers, such as platelet-endothelial cell adhesion molecule-1 (CD31), Cdh5 (vascular endothelial cadherin), and von Willebrand factor (Figure 1) [28, 29].

Because the characterization of EPCs is controversial, additional criteria for defining EPCs, based on morphology and culture procedures, have been established (Table 1). To isolate and expand EPCs from umbilical cord and peripheral blood mononuclear cells, three culture methods have been described [31]. The first involves culturing mononuclear cells on fibronectin-coated dishes and replating the nonadherent cells after 48 h. In that method, angiogenic cells arise after 4–9 days as round cells surrounded by spindle-shaped cells. The second method involves culturing mononuclear cells on fibronectin-coated dishes, in this case for 4 days, and keeping the adherent cells, which give rise to a heterogeneous population of cells termed circulating angiogenic cells. These cells do not form colonies or express endothelial cell surface antigens but do retain the characteristics of monocytes. The exact origin and role of these cells* in vivo* are a question that remains unanswered, and they may come from detachment of endothelium layer cells, mesenchymal stem cells, or hematopoietic stem cells [27]. The third culture method involves a longer period of mononuclear cell culture on collagen-coated dishes. Between days 7 and 21, a population of cells originates from the adherent cells, and that population has been characterized as being composed of endothelial outgrowth cells [32, 33]. Colonies of endothelial outgrowth cells are identified by their cobblestone-like structure [14, 34] and are cultivated in medium supplemented with growth factors such as epidermal growth factor, VEGF, basic fibroblast growth factor (bFGF), insulin-like growth factor-1, ascorbic acid, and a gentamicin-amphotericin B mix. However, it has been shown that the presence of mesenchymal stem cells induces EPCs to differentiate into endothelial cells, promoting angiogenesis even without the addition of exogenous growth factors [35], indicating the important roles that paracrine effects and direct cell contact of these cell subtypes play in the modulation of the angiogenic response. Bone marrow-derived EPCs have also been isolated from mononuclear cell phase or whole bone marrow cell extract and cultivated in fibronectin-coated dishes for 7 days. After two days of culture, a small “blood island” appears, and cells adhere to the plate, exhibiting spindle-like appearance, after seven days [36].

It seems that the differentiation of endothelial cells and their maturation as vascular cells, together with the formation and stabilization of new blood vessels, constitute a dynamic and complex process that comprises different types of cells, each of which has a specific function and all of which are essential to the final result. Although the phenotype and characterization of EPCs have yet to be fully determined, their origins and functions have been well established in the last decade [2–5, 21–27]. The challenge now is to understand the mechanisms involved in the formation of new blood vessels and how they lead to benefit or harm.

## 3. Molecular Signaling in Angiogenesis

Vasculogenesis is defined as the formation of the primitive vasculature network during the embryonic period, whereas angiogenesis is defined as the formation of new blood vessels from preexisting vasculature. However, because adult EPCs have now been identified, the best term to describe this complex process in which mature and progenitor endothelial cells take part would be postnatal vasculogenesis. Although the formation of new blood vessels is vital to many beneficial physiological processes, such as wound healing and bone repair [37, 38], it can also be involved in pathological conditions, including arthritis [39] and diabetic retinopathy [40], as well as tumor growth and metastasis [41]. Therefore, angiogenesis is largely studied as a target of new therapeutic strategies.

Angiogenesis and its role in tumor growth were first described in the 1970s by Ausprunk and Folkman [42]. It is now known that angiogenesis involves two separate processes. The process described by Ausprunk and Folkman [42], designated sprouting angiogenesis, is characterized by migration, proliferation, three-dimensional organization, and tube formation of endothelial cells. More recently, another process, known as nonsprouting angiogenesis (intussusception), has been described and is defined as the division of vessels by transluminal pillar formation through invagination with interstitial tissue [43].

The induction of angiogenesis relies on a tenuous balance between pro- and antiangiogenic factors. The proangiogenic factors include bFGF [44], platelet-derived growth factor [45], platelet-derived endothelial cell growth factor [46], angiopoietin-1 [47], transforming growth factor beta-1 [48], transforming growth factor alpha, and epidermal growth factor [49]. However, the most well-known proangiogenic factor is VEGF-A. Other VEGF family subtypes, such as VEGF-B, VEGF-C, VEGF-D, and placental growth factor, have also been shown to be involved in tissue-specific forms of angiogenesis, including myocardial angiogenesis [41], embryonic angiogenesis, and lymphangiogenesis. In addition, VEGF-A stimulates mitogenesis and cell migration, as well as increasing vasodilatation and vascular permeability. These effects are mediated by activation of tyrosine kinase receptors (VEGFRs), which are present on the cell surface.

It has been demonstrated that VEGF-A binds to VEGFR-1 and VEGFR-2 [50]. Most of the biological effects of VEGF-A are mediated by VEGFR-2, whereas VEGFR-1 activation is still not completely understood. Despite the fact that VEGF-A binding to VEGFR-1 is 10-fold higher than VEGF-A binding to VEGFR-2 [51], some evidence suggests that VEGFR-1 mediates angiogenesis during embryogenesis [52], whereas the architectural organization of new blood vessels without mitogenic activity is controlled by VEGFR-2 [53]. In addition, because of the high VEGFR-1 binding affinity without activation of downstream signaling, VEGFR-1 is considered to be a trap receptor, making VEGF-A less available to VEGFR-2 binding [54]. Other receptors for different isoforms of VEGF were also described. Neuropilin-1 and neuropilin-2 were originally identified as receptor for axon guidance factors belonging to semaphorins family. Subsequently, the binding of VEGF isoforms to these receptors revealed their participation in the angiogenesis modulation [55]. VEGF165 isoform binds to neuropilin-1 and increases the proliferation and migration of cells that express VEGFR2 [56], suggesting that neuropilins may interact with VEGFRs contributing to physiological and pathological angiogenesis [57]. In addition, semaphorins have also been shown to favor tumor growth by promoting angiogenesis [58].

Activation of VEGFR-2 induces various intracellular signaling pathways. After VEGF-VEGFR-2 binding, tyrosine phosphorylation activates phospholipase C, thus increasing inositol triphosphate levels, which leads to Ca^2+^ fluxes. This process also generates diacylglycerol, thereby activating protein kinase C and inducing activity of extracellular-signal regulated kinases (ERK) 1 and 2, resulting in proliferation [50]. In addition, VEGFR-2 binds to phosphoinositide 3-kinase, which has been implicated in the tube formation, proliferation, survival, and vascular permeability of endothelial cells [59]. These are the signals that contribute to angiogenesis promotion, and the inhibition of this pathway has been the object of numerous studies aimed at increasing the efficacy of antiangiogenic treatment strategies in patients with tumors. Although most such studies have focused on therapies that block VEGF signaling, there are other factors that regulate the VEGF pathways.

Various products of the alternative splicing of VEGF-A mRNA have antiangiogenic properties. A shift in the balance of alternative splicing (toward expression of pro- or antiangiogenic VEGF isoforms) is modulated by physiological or pathophysiological processes [60]. Other molecules, such as semaphorin 3E, which binds to the neuropilin receptor, are also involved in the inhibition of angiogenesis [61]. Therefore, in addition to understanding how to inhibit angiogenic factors, it is thought to be important to establish an antiangiogenic balance in order to create the conditions that would allow new therapeutic strategies to inhibit or stimulate angiogenesis.

Hypoxia (the loss of vascular function leading to low oxygen tension) is the main trigger for the elaborate process of angiogenesis. Damaged tissue and tumor tissue both present hypoxic environments. Various mechanisms are triggered when there is a need to restore the supply of oxygen. The most important is the activation of transcription factor hypoxia-inducible factor-1 (HIF-1), which induces the expression of adhesion molecules [62], matrix components [63, 64], metabolic proteins [65], and growth factors, such as VEGF-A [66, 67]. Thus, physiological and pathological angiogenesis both result from an imbalance between pro- and antiangiogenic factors, favoring the former.

Once VEGF-A is expressed, it becomes the major player in angiogenesis. Produced by a variety of different cell types, such as macrophages, platelets, retinal epithelial cells, tumor cells, and endothelial cells, VEGF-A has antiapoptotic effects and is a potent mitogen. It also stimulates the production of adhesion molecules [62], matrix components [63, 64], and matrix metalloproteinases (MMPs) [65]. The main targets of VEGF-A are endothelial cells, where it stimulates the production and release of nitric oxide (NO), which causes local vasodilatation. In addition, after the release of nitric oxide, endothelial cells change their shape and cell-to-cell adherence is reduced, resulting in increased vascular permeability, which allows circulating cells and proteins to reach the site of injury. Nevertheless, some tissues have no capacity to regenerate after an injury because they are avascular. It has been demonstrated that the use of VEGF-A in the treatment of tears in the medial menisci, whose lateral two-thirds are avascular, does not promote tissue repair because there is no formation of a complex vascular bed [66]. These data suggest that angiogenesis relies on a network of complex events rather than VEGF-A stimulation alone. Furthermore, most avascular tissues have an intrinsic mechanism that blocks this process. Therefore, angiogenesis not only requires a shift from an antiangiogenic to a proangiogenic balance but also depends on other concurrent events that do not always take place.

Unlike the vascularization that occurs during tissue repair, the vascularization of tumors is characterized by a chaotic network composed by vessels of different calibers and often with blind ends. One explanation for this characteristic is that HIF-1 expression promotes the constant release of bFGF and VEGF-A, recruiting cells to the site of vessel formation in an unregulated manner [67].

## 4. EPCs in Cancer Angiogenesis

Although the molecular mechanisms involved in the activation of angiogenesis are well understood, many antiangiogenic therapies have failed due to alternative molecular escape routes [68]. In this context, another important point to be considered is the cellular component of the angiogenesis process. Until the last decade, it was thought that angiogenesis was promoted only by stimulation of mature endothelial cells. Recent studies have demonstrated the role played by EPCs. Ischemic stimuli are often sufficient to recruit EPCs from bone marrow [2, 8, 24, 69], after which they can incorporate into sites of injury and promote vascularization. Additional evidence of EPC-related protection in vascular diseases comes from the finding that there is an inverse correlation between the number and quality of circulating EPCs in the peripheral blood of patients with cardiovascular impairment [7, 70–74].

It has been demonstrated that EPCs migrate to sites of vascular damage, such as an ischemic limb, an injured retina, and an infarcted myocardium, where they increase vascularization, as well as improving blood flow and tissue performance [8, 26, 69, 75]. Studies of treatment with mature endothelial cells and EPCs have shown that the latter induce neovascularization after myocardial infarction [8, 76], suggesting that EPCs reduce ischemic damage by promoting angiogenesis and that mature endothelial cells do not.

Despite the knowledge that EPCs are a feasible treatment for a variety of clinical conditions, it remains unclear which specific type of EPC would be most appropriate for use in stem cell therapy. The great majority of the studies on the topic have failed to determine whether the injected cells were angiogenic cells or endothelial outgrowth cells, and the initial characterization of the cells has often been incomplete. Although the injection of only one type of EPC induces neovascularization [14, 77], treatment with both types promotes significantly greater tissue repair [14]. Therefore, bidirectional communication between angiogenic cells and endothelial outgrowth cells appears to be important for improving physiological angiogenesis. However, the chaotic tumor microenvironment leads to dysfunction of angiogenic stimuli and formation of a disorganized vascular network. Many cancers, such as some forms of leukemia, lymphoma, and breast cancer [78], have been associated with an increase in the number of circulating EPCs. In addition, EPC recruitment favors tumorigenesis. In a study involving xenotransplantation of human tumors in mice, the size of the tumors was found to increase when EPCs were injected systemically, which resulted in better vascular network formation within the tumor microenvironment [28].

Tumors produce growth factors that modulate angiogenesis, including VEGF-A and bFGF. The release of such growth factors and the hypoxic tumor microenvironment recruits EPCs from bone marrow or activates tumor residents EPCs. Those EPCs either differentiate into endothelial cells or produce angiogenic growth factors. In brief, tumor hypoxia favors the rupture of the extracellular matrix (ECM) due to the release of MMPs, which contribute to tumor angiogenesis and metastasis. In addition, within the tumor microenvironment, mesenchymal stem cells and other cells, including pericytes, can constitute an additional source of proangiogenic factors, playing an important role in covering and protecting newly formed vessels. The release of VEGF-A not only induces tumor angiogenesis but also inhibits the recognition and destruction of tumor cells by the immune system [79]. Neovascularization ensures an adequate supply of oxygen and blood for tumor progression, as well as facilitating metastasis, because it provides a route by which tumor cells get into the bloodstream and spread throughout the system, an increase in vascular density having been shown to increase metastatic potential [80].

**Figure 2:**
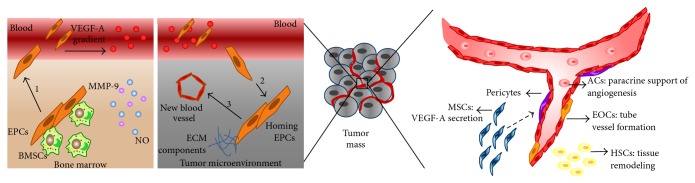
EPCs are the major players in new vessel formation contributing to tumor growth and metastasis. They might be recruited from bone marrow and migrate to the tumor (on the left) or either reside within tumor stroma, where there are other stem/progenitor cells that promote and/or contribute to new vessel formation (on the right: ACs: angiogenic cells; BMSCs: bone marrow-derived stem cells; ECM: extracellular matrix; EOCs: endothelial outgrowth cells; EPCs: endothelial progenitor cells; HSCs: hematopoietic stem cells; MMP: metalloproteinase; MSCs: mesenchymal stem cells; NO: nitric oxide; VEGF: vascular endothelial growth factor). Figure adapted from Melero-Martin and Dudley [30] (license number 3593771424850).

The role of EPCs in promoting tumor angiogenesis and metastasis has been the target of many studies aimed at developing new therapeutic strategies. However, identifying the origin of these cells and determining their exact location after injection continue to pose challenges. Some authors believe that hematopoietic stem cells, mesenchymal stem cells, and EPCs reside in the stroma surrounding the tumor mass. Melero-Martin and Dudley [30] showed that paracrine crosstalk between tumor cells and stromal cells results in an unexpected pattern of stem/progenitor cell differentiation, which could accelerate the progression of the tumor. The authors argued that whereas EPCs are the primary agents of lumen formation in new angiogenic sprouts, mesenchymal stem cells and hematopoietic stem cells act as angiogenic stimulators by secreting VEGF-A, as well as tissue remodeling and endothelial survival factors that sustain the process of angiogenesis. Mesenchymal stem cells can also differentiate into pericytes, which support the formation of new blood vessels. Therefore, these three populations of stem/progenitor cells work in concert to form the building blocks of tumor vascularization (Figure 2).

The overarching question is how the activation of stem/progenitor cells present in the stroma of a tumor is coordinated. One hypothesis is that ECM remodeling around the tumor mass can provide signals to the stem/progenitor cells regarding their differentiation and plasticity [81]. Because ECM compounds are important to EPC growth and differentiation [82, 83], disruption of the ECM by tumor cells can lead to activation of the “incorrect” EPC fate. A second clue to the deregulation of the fate of stem/progenitor cells is epigenetic alterations caused by their interaction with the tumor microenvironment. Extracellular factors released by tumor cells regulate epigenetic alterations in cells within the stroma of the tumor, promoting its growth and metastasis. In a study of prostate cancer, changes in the epigenetic patterns of endothelial cells in the tumor microenvironment showed that methylation of the promoter of the gene* CYP24A1* plays a role in determining the phenotype of the tumor-associated vasculature [84]. In addition, hypermethylation of a specific tumor suppressor gene in mesenchymal stem cells has been shown to cause those cells to display various features of cancer stem-like cells/cancer-initiating cells, including loss of anchorage dependence, increased colony formation capability, drug resistance, and pluripotency [85]. These stromal cells might coevolve with tumor cells during tumor progression, acquiring the characteristics of nearby tumor cells, thus contributing to the formation of new blood vessels.

It seems that VEGF-A mediates not only tumor angiogenesis but also the maintenance of the stem cells surrounding the tumor. Beck et al. [86] showed that blocking VEGFR-2 results in tumor regression because it decreases microvascular density and reduces the size of the stem cell pool, thereby impairing their renewal capacity. Those authors identified a dual role for tumor cell-derived VEGF-A in promoting cancer stemness: by stimulating angiogenesis in a paracrine manner, thus creating a perivascular niche for stem cells, and by directly affecting stem cells in an autocrine loop, thus stimulating cancer stemness and renewal.

Unlikely, Melero-Martin and Dudley [30] and Nolan et al. [87] proposed a different origin for cancer EPCs. They demonstrated that bone marrow-derived EPCs are the main source of endothelial cells that could contribute to neovascularization, mainly in early tumors [86]. In addition to vascular tube formation in tumors [88, 89], bone marrow-derived EPCs have also been shown to participate in a paracrine fashion by exerting self-enhancement effects and regulating the expression of interleukin-1 beta in THP-1 monocytes [90], as well as that of monocyte chemotactic protein-1 in hepatocellular carcinoma [91]. Therefore, the success of tumor angiogenesis promoted by bone marrow-derived EPCs depends on the occurrence of three consecutive events: recruitment of the EPCs from the bone marrow to the peripheral blood, EPC homing to and invasion of the tumor site, and EPC differentiation into mature endothelial cells for the formation of new blood vessels (Figure 2). The recruitment of EPCs from bone marrow is regulated by a variety of growth factors, enzymes, ligands, and membrane receptors. The most important factor in the recruitment of EPCs is VEGF-A, which, upon stimulation by a protease, typically MMP-9 induced by endothelial nitric oxide synthase activity, allows the detachment of EPCs and their release into the systemic circulation. The release of EPCs is regulated by stromal cell-derived factor-1, also known as chemokine (CXC motif) ligand 12 (CXCL12), and its receptor, chemokine (CXC motif) receptor 4 (CXCR4), as well as by bone marrow integrins [92–94].

The migration of EPCs to the site of a tumor occurs by chemokine gradients that activate their correspondent cell receptors. The main participants are VEGF-A/VEGFR-2, CXCL12/CXCR4, growth-regulated oncogene alpha/CXCR1, interleukin-8/CXCR2, chemokine (C-C motif) ligand 2 (CCL2)/chemokine (C-C motif) receptor 2 (CCR2), and CCL5/CCR5 [78], and it seems that they act mutually, because CXCL12 expression depends on the quantity of VEGF-A [95, 96]. Once in the tumor bed, EPCs interact with endothelial cells via selectins, integrins, and adhesion molecules, allowing adhesion and migration to the site where new vascularization is needed [78]. After transendothelial migration and tissue homing, EPCs interact with ECM compounds to induce cell differentiation. Fibronectin is the major factor promoting VEGF-induced differentiation of EPCs in mature endothelial cells [97]. Therefore, crosstalk among endothelial cells, pericytes, and hematopoietic stem cells favors ECM remodeling to support the maturation and stabilization of the network of capillary tubes [98].

In general, there are two schools of thought regarding tumor angiogenesis promoted by EPC activation: one that promotes the idea that stem/progenitor cells reside in the tumor microenvironment and that certain factors disrupt their fate and stemness and the other that subscribes to the idea that EPCs are recruited from the bone marrow to the tumor site. Based on their intrinsic ability to home to tumor sites, EPCs are attractive as cell vectors for targeted cancer gene therapy. Scientists have developed genetically engineered EPCs, transfected with vectors encoding some specific antitumor molecules. In that approach, the cells retain their homing properties but lose their capacity to form new blood vessels. In animal models of melanoma, delivery of specific MMP-12 by such EPCs has been shown to induce cleavage of molecules that induce tumor progression, thus inhibiting tumor growth, angiogenesis, and metastasis [99]. The same type of therapy has also prolonged the survival in tumor-bearing mice. The genetically modified EPCs release CD40 ligand that induces the production of tumor necrosis factor and interferon gamma, as well as increasing the activity of caspase-3 and caspase-7, in metastatic lung cancer [100]. In addition, it has been demonstrated that these cells enhance antitumor effects by inhibiting angiogenesis and inducing apoptosis in a murine model of glioma [101].

## 5. Conclusion

In the promotion of angiogenesis, EPCs are crucial, and they are attracted to hypoxic environments. Characterizing the various types of EPCs is hard work, and there have been some studies aimed at clarifying the distinctions among them. Some cell markers, such as CD45 and CD14, might be useful in distinguishing endothelial outgrowth cells, which differentiate into mature endothelial cells, being the real building blocks of the vessel walls, from angiogenic cells, which are derived from hematopoietic stem cells producing diverse cytokines that initiate and maintain the angiogenesis cascade.

Angiogenic cells and endothelial outgrowth cells have both been used in the treatment of ischemic diseases, either in animal studies or in clinical trials, and favorable results have been obtained. When the two cell types have been used in combination, therapeutic goals have been met in a more satisfactory manner, indicating that they act in concert. Despite the beneficial effect of EPC-induced angiogenesis during wound healing, such angiogenesis is actually harmful when it occurs in a tumor, contributing to its growth and metastasis. However, because of ability of EPCs to migrate and home to a tumor site, their use as a vector in gene therapies has become a promising therapeutic strategy. In this review, the main role of EPCs in the angiogenesis process was described. A better understanding of the molecular biology of EPCs could facilitate the identification of new targets for cancer therapy.

## Figures and Tables

**Table 1:** Main characteristics distinguishing endothelial outgrowth cells and angiogenic cells.

Characteristic	Endothelial outgrowth cells	Angiogenic cells
Time for culture growth	7–21 days	3–5 days
Morphology	Confluent cobblestone monolayer	Round to spindle-shaped
Surface antigens	CD34^+^ VEGFR-2^+^ CD45^−^ CD14^−^ CD133^−^	CD34^low^ VEGFR-2^low^ CD45^+^ CD14^+^
Nitric oxide production	High	Low
Cytokine secretion	Low	High
Vascular tube formation	Generate vascular tubes in Matrigel	Do not generate vascular tubes in Matrigel
Neovascularization capacity	Improve neovascularization	Improve neovascularization
Cell properties	Bind UEA-I lectin and take up LDL	Bind UEA-I lectin and take up LDL

VEGFR: vascular endothelial growth factor receptor; UEA-I: *Ulex europaeus* agglutinin I; LDL: low-density lipoprotein.
